# Genetic Diversity and Population Structure of Largefin Longbarbel Catfish (*Hemibagrus macropterus*) Inferred by mtDNA and Microsatellite DNA Markers

**DOI:** 10.3390/ani15060770

**Published:** 2025-03-08

**Authors:** Yanling Hou, Huan Ye, Huamei Yue, Junyi Li, Ling Huang, Ziling Qu, Rui Ruan, Danqing Lin, Zhiqiang Liang, Yong Xie, Chuangju Li

**Affiliations:** 1 College of Fisheries and Life Sciences, Shanghai Ocean University, Shanghai 201306, China; 2 Key Laboratory of Freshwater Biodiversity Conservation, Ministry of Agriculture and Rural Affairs, Yangtze River Fisheries Research Institute, Chinese Academy of Fishery Sciences, Wuhan 430223, China; 3 Key Laboratory of Freshwater Fisheries and Germplasm Resources Utilization, Ministry of Agriculture and Rural Affairs, Freshwater Fisheries Research Center, Chinese Academy of Fishery Sciences, Wuxi 214081, China; 4 Hunan Fisheries Science Institute, Changsha 410153, China; 5 Chongqing Fishery Sciences Research Institute, Chongqing 400020, China

**Keywords:** largefin longbarbel catfish, mitochondrial cytochrome *b* (*Cytb*), microsatellite, conservation genetics

## Abstract

The largefin longbarbel catfish (*Hemibagrus macropterus*), a freshwater species endemic to China with fundamental economic importance, requires investigation into its genetic structure for effective management. In this study, we employed mitochondrial cytochrome *b* (*Cytb*) gene sequences and 14 microsatellite loci to elucidate the genetic structure of 195 individuals across eight distinct populations. The *Cytb* analysis revealed a haplotype number (H) of 31, haplotype diversity (Hd) of 0.853, and nucleotide diversity (π) of 0.0127. Population neutrality tests indicated that Tajima’s D (−0.59467) and Fu and Li’s D* (0.56621) were not statistically significant, and the mismatch distribution exhibited a multimodal pattern. Microsatellite analysis revealed that the mean number of alleles (Na), observed heterozygosity (Ho), and polymorphic information content (PIC) across all loci were 18.500, 0.761, and 0.808, respectively. The UPGMA phylogram constructed based on genetic distance identified two distinct clusters, with paired Fst values ranging from 0.108 to 0.138. These results suggest that the largefin longbarbel catfish is in a state of dynamic equilibrium with high genetic diversity. Furthermore, there was significant genetic differentiation between the YB population and the other seven populations, indicating that the population in the upper reaches of the Yangtze River should be managed as a distinct unit.

## Simple Summary

Understanding the genetic background of populations is crucial for their commercial exploitation and conservation. This study analyzed the genetic diversity and population structure of seven wild populations and one stock seed population of the largefin longbarbel catfish (*Hemibagrus macropterus*). The genetic diversity parameters indicated that the largefin longbarbel catfish maintains high genetic diversity. Furthermore, significant genetic differentiation was observed between the population in the upper reaches of the Yangtze River and the other populations. These findings provide essential genetic information for the development of genetic breeding programs and the conservation of wild resources of the largefin longbarbel catfish.

## 1. Introduction

The largefin longbarbel catfish (*Hemibagrus macropterus*), a member of the Bagridae family comprising over 220 species [1], is an endemic, medium-sized benthic fish native to China, predominantly found in the Yangtze and Pearl River basins [2]. This species is highly valued for its tender flesh, minimal bone spurs, and significant nutritional benefits, making it an economic fish with substantial demand, particularly in Southwest China [3], where the market price is as high as CNY 100–200 per kilogram. This species was assessed as Least Concern according to the IUCN Red List of Threatened Species in 2011, nonetheless, recent years have witnessed a decline in its wild populations due to anthropogenic activities such as overfishing, water pollution, habitat destruction, and dam construction, which have collectively resulted in a reduction in breeding individuals, degradation of habitats and spawning sites, and a consequent sharp decline in its natural resources. To address the burgeoning market demand, researchers have been engaged in developing artificial propagation techniques for the largefin longbarbel catfish since the 1990s, achieving notable advancements in recent times [4,5,6]. Currently, the majority of largefin longbarbel catfish available in the Chinese market come from commercial aquaculture with an annual output of dozens of tons, and the breeding scale reaches several hundred thousand. Meanwhile, a portion of these artificially bred individuals are utilized for release into the wild as part of efforts to restore the natural resources of largefin longbarbel catfish. During the domestication process, intense artificial selection and the insufficient introduction of new parental stock have resulted in a limited number of parental individuals sustaining the population. This scenario potentially gives rise to reduced genetic diversity and the accumulation of deleterious genetic variations, resulting in the degradation of germplasm resources [7].

Genetic diversity, or polymorphism, arises from the variation in genetic material among individuals within the same species [8]. It is fundamental to the adaptability of populations, equipping them with the capacity to confront survival challenges [9,10]. The advent and advancement of molecular biology have introduced a range of genetic markers, such as mitochondrial DNA (mtDNA) [11], random amplified polymorphic DNAs (RAPDs) [12], restriction fragment length polymorphisms (RFLPs) [13], amplified fragment length polymorphisms (AFLPs) [14], simple sequence repeats (SSRs) [15], and single nucleotide polymorphism (SNP) [16], which are extensively utilized in genetic research across various organisms, significantly advancing the field of population genetics [17,18].

Due to its straightforward structure, matrilineal mode of inheritance, moderate rate of evolution, and sensitivity to phylogenetic information [19], mtDNA has been extensively employed in the investigation of genetic diversity and the historical dynamics of populations, as exemplified by studies on *Megalobrama skolkovii* [19], *Acrossocheilus* [20], and *Hemibagrus guttatus* [21]. Furthermore, SSRs are widespread across the genomes of both prokaryotic and eukaryotic organisms, comprising tandem repeats of 1–6 nucleotides [22]. This marker is characterized by their high informational content, locus specificity, significant intraspecific polymorphism, and co-dominant inheritance. As a result, they have been widely utilized in research concerning genetic diversity, population genetic structure, molecular breeding, paternity testing, and gene flow assessment in aquatic species, such as *Hypophthalmichthys molitrix* [23] and *Pelteobagrus fulvidraco* [24]. The integration of mtDNA and SSRs has been utilized to investigate the genetic structure of numerous aquatic organisms within the Yangtze River, including *Leiocassis longirostris* [25], *Coilia* [26], and *Eriocheir sinensis* [27]. However, in the case of the largefin longbarbel catfish, research has been limited to a single study conducted in 2009, which employed the *Cytb* gene to analyze the genetic structure and geographical differentiation among 12 populations comprising 96 specimens [28].

The genome of the largefin longbarbel catfish has been assembled at the chromo-some level (CNGBdb accession No. PRJCA019452) [29], thereby offering valuable genetic resources for research. However, its genetic structure has yet to be investigated using genomic approaches. Therefore, a comprehensive study is necessary to elucidate the population structure of the largefin longbarbel catfish, which is essential for informing breeding programs and the conservation of wild resources.

In this study, a fragment of *Cytb* gene and 14 microsatellite markers were employed to assess the genetic diversity and population structure of seven wild populations and one seed stock population of the largefin longbarbel catfish. The findings are anticipated to offer valuable insights for artificial breeding programs, artificial release initiatives, and the recovery and management of wild populations.

## 2. Materials and Methods

### 2.1. Sample Collection and DNA Extraction

**Figure 1:**
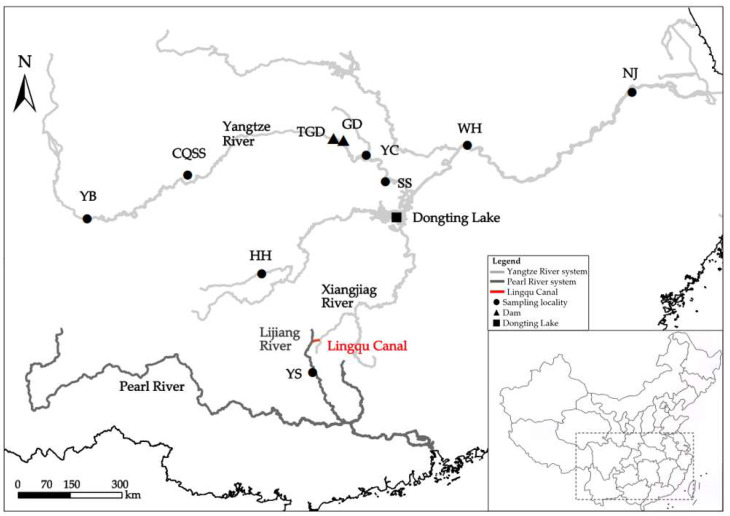
Sampling localities for *Hemibagrus macropterus* populations. YB, Yibin; YC, Yichang; SS, Shishou; WH, Wuhan; NJ, Nanjing; HH, Huaihua; YS, Yangshuo; CQSS, Stock seed; TGD, Three Gorges Dam; GD, Gezhouba Dam.

A total of 195 specimens were collected from seven distinct localities within the Yangtze River and Pearl River basins, as well as from a seed stock population at the Largefin Longbarbel Catfish Foundation Seed Farm, affiliated with the Fisheries Science Institute of Chongqing (Figure 1, Table 1). Fin rays, preserved in 100% ethanol, or blood samples were obtained from fresh specimens and subsequently stored at −20 °C. Genomic DNA was extracted from each specimen using the TaKaRa MiniBEST Universal Genomic DNA Extraction Kit Ver.5.0 (TaKaRa, Kusatsu City, Shiga Prefecture, Japan). The concentration and quality of the extracted DNA were assessed using a NanoDrop 8000 spectrophotometer (Thermo Fisher Scientific, Carlsbad, CA, USA) and 1% agarose gel electrophoresis, respectively. The DNA was then stored at −20 °C for future experimental procedures.

### 2.2. Amplication of Cytb Gene Sequence in Largefin Longbarbel Catfish

Universal primers L 14724 (5′-GACTTGAAAAACCACCGTTG-3′) and H 15915 (5′-CTCCGATCTCCGGATTACAAGAC-3′) were applied to amplify the *Cytb* gene fragment via PCR, as described in previous studies [30]. The PCR mixture comprised 2 × Taq PCR Master Mix (Vazyme Biotech Co., Ltd., Nanjing, China), 20 ng of DNA template, and 10 pmol of each primer, with a total reaction volume of 20 μL. PCR amplification was conducted under the following conditions: an initial denaturation at 95 °C for 5 min, followed by 35 cycles of amplification (95 °C for 30 s, 60 °C for 30 s, 72 °C for 30 s), and a final extension at 72 °C for 5 min. The quality of each PCR product was evaluated by 1% agarose gel electrophoresis, followed by sequencing on an ABI3730xl DNA analyzer (Applied Biosystems, Inc., Carlsbad, CA, USA).

### 2.3. Microsatellite Amplification and Typing in Largefin Longbarbel Catfish

SSR analysis was conducted on the transcriptome data of the largefin longbarbel catfish, resulting in the selection of 200 SSR loci. Primers for these loci were designed using Primer Premier v5.0 [31]. The specificity, stability, and polymorphism of the loci were evaluated by randomly selecting 10 individuals, leading to the identification of suitable loci. Forward primers labeled with fluorescent dyes (TAMRA, FAM, or HEX) at the 5′ end were utilized for PCR amplification to produce fluorescently labeled PCR products. The PCR amplification was executed using the VeritiPro™ Thermal Cycler, 384-well (Thermo Scientific, Waltham, MA, USA), with a reaction mixture containing 1 µL of sample DNA (~20 ng), 5 µL of 2 × Taq Master Mix (Vazyme), 0.5 µL of each primer, and 3 µL of double-distilled water. The thermal cycling conditions were as follows: an initial denaturation at 95 °C for 5 min; followed by 35 cycles of 30 s at 95 °C, 30 s at 52 °C to 62 °C, and 30 s at 72 °C; with a final extension at 72 °C for 10 min. To ensure primer specificity and uniformity of the PCR product, 2 µL of the PCR product was subjected to agarose gel electrophoresis at a 1% concentration following PCR amplification. The efficiency and specificity of amplification for each primer were assessed based on the intensity and pattern of the bands, respectively. PCR products were analyzed using an ABI 3730xL DNA Analyzer (Applied Biosystems), and the resulting data were processed with GeneMarker^®^ HTS v 2.5.0 software [32].

### 2.4. Data Analysis of Cytb in Largefin Longbarbel Catfish

The genetic diversity indices of the *Cytb* gene, including the number of sequences, number of haplotypes (h), haplotype diversity (Hd), and nucleotide diversity (π), were evaluated using DNAsp v5.0 [33]. Genetic distances between different populations were calculated using the Kimura-2-parameter distance model (K2P) in MEGA v7.0 software [34]. A network of haplotype relationships was constructed using the Median-Joining model [35]. Analysis of molecular variance (AMOVA) was conducted to determine the distribution of genetic variation within and among populations, and pairwise genetic differentiation indices (Fst) were calculated between populations using Arlequin v3.5 software [36]. Neutrality tests were performed using Tajima’s D and Fu’s Fs statistics [37].

### 2.5. Data Analysis of SSR in Largefin Longbarbel Catfish

The polymorphic information content (PIC) of each locus and the genetic diversity indices for each population, including the number of observed alleles (Na), number of effective alleles (Ne), observed heterozygosity (Ho), and expected heterozygosity (He), were calculated using the software packages Cervus v3.0 [38], GenAlEx v6.501 [39], or Genepop v4.0 [40]. Nei’s genetic distance (Nei, 1983) among all populations was calculated from allele frequencies using PowerMarker v3.25, and based on the Nei’s genetic distance matrix, cluster analysis was conducted employing the unweighted pair group method with arithmetic means method (UPGMA). To evaluate genetic variation within individuals and populations, as well as between populations, analysis of molecular variance (AMOVA) was performed, and paired genetic differentiation coefficients were calculated. Principal Coordinate Analysis (PCoA) was utilized to illstrate the similarities or differences among related populations, using GenAlEx v6.501 and Arlequin v3.5. Genetic clustering patterns were assessed using a Bayesian model implemented in STRUCTURE v2.3.4 [41]. The value of K was set to vary from 1 to 20. For each value of K, analyses were conducted 20 times, incorporating a burn-in period of 10,000 iterations followed by 100,000 iterations of the Markov Chain Monte Carlo (MCMC) method. Subsequently, the ΔK value was computed to ascertain the most appropriate number of genetic clusters, as indicated by the ΔK metric.

## 3. Results

### 3.1. Genetic Diversity

#### 3.1.1. Genetic Diversity of *Cytb* in Largefin Longbarbel Catfish

A total of 31 haplotypes were identified from 195 individuals based on the *Cytb* gene (Table 2). The haplotype diversity (Hd) varied from 0.154 in the YB population to 0.887 in the HH population, with an overall haplotype diversity of 0.853. Nucleotide diversity (π) ranged from 0.0005 in the YS population to 0.0108 in the YC population, with an overall nucleotide diversity of 0.0127 (Table 3). The number of haplotypes per population ranged from 2 to 15, with the YB population exhibiting the fewest haplotypes (h = 2) and the HH population displaying the most (h = 15), suggesting that the HH population possesses the highest genetic diversity, whereas the YB population exhibits the lowest genetic diversity.

#### 3.1.2. SSRs Polymorphism and Genetic Variation in Largefin Longbarbel Catfish

Following the detection of primers, 14 SSR loci capable of stable amplification were identified (Table 4). Utilizing these 14 pairs of microsatellite primers, a total of 259 alleles were detected across 195 individuals from eight distinct populations of the largefin longbarbel catfish. The number of alleles (Na) ranged from 9 (Hem002) to 44 (Hem041), with an average of 18.500. The effective number of alleles (Ne) varied from 2.599 (Hem002) to 13.787 (Hem041), with an average of 7.488. Observed heterozygosity (Ho) ranged from 0.495 to 0.928, with an average value of 0.761, while expected heterozygosity (He) varied from 0.615 to 0.927, with an average of 0.828. All 14 microsatellite markers exhibited high levels of polymorphism, as indicated by polymorphic information content (PIC) values ranging from 0.557 to 0.923, with an average of 0.808 (Table 5).

The number of alleles (Na) across all populations ranged from 6.786 in the YB population to 11.857 in the HH population, while the effective number of alleles (Ne) ranged from 4.393 in the YB population to 7.457 in the HH population. Observed heterozygosity (Ho) varied from 0.697 in the YB population to 0.830 in the HH population, and expected heterozygosity (He) ranged from 0.706 in the CQSS population to 0.831 in the HH population (Table 6).

### 3.2. Genetic Differentiation and Population Structure

#### 3.2.1. Genetic Differentiation and Population Structure Based on *Cytb*

The genetic distance among the eight populations varied from 0.0624 (YB/HH) to 0.0014 (SS/WH), while pairwise Fst values ranged from −0.0333 (YC/CQSS) to 0.9137 (YB/YS) (Table 7). Among those, the HH and YB populations exhibiting significant genetic differentiation from other populations (Fst > 0.25). The AMOVA analysis of the *Cytb* gene indicated that 65.65% of the genetic variation was attributable to differences among populations, whereas 34.35% occurred within populations, suggesting that the genetic differences primarily originated from inter-population variation (Table 8).

**Figure 2:**
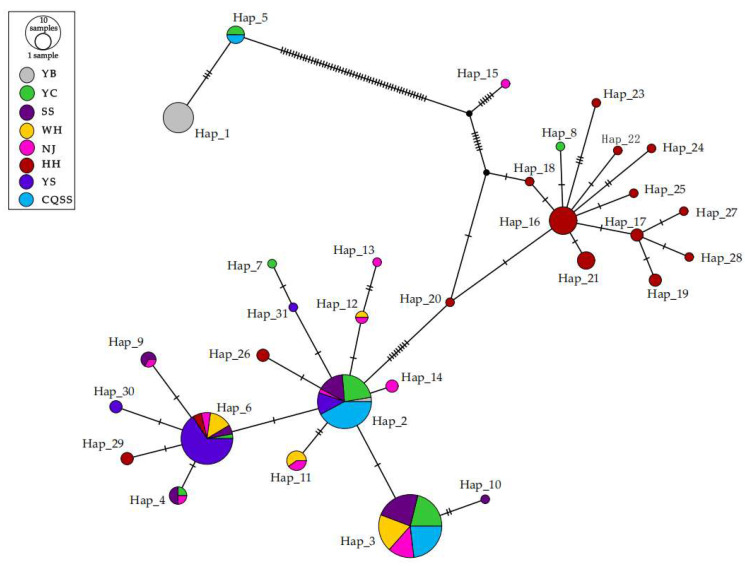
Haplotype network diagram of eight populations of *Hemibagrus macropterus* based on *Cytb*. YB, Yibin; YC, Yichang; SS, Shishou; WH, Wuhan; NJ, Nanjing; HH, Huaihua; YS, Yangshuo; CQSS, Stock seed.

Network analysis illustrated the frequency and distribution of *Cytb* gene haplotypes (Figure 2). Haplotype 2 and Haplotype 6 emerged as the most prevalent, both appearing in six populations. The YS population exhibited four distinct haplotypes, two of which were shared with populations in the middle and lower reaches of the Yangtze River, indicating a relatively close genetic relationship between the Pearl River population and those in the middle and lower Yangtze River. HH population may have spread from the populations in the middle reaches of Yangtze River, and the diffused population had differentiated into new haplotypes.

#### 3.2.2. Genetic Differentiation and Population Structure Based on SSR

The result of genetic differentiation analysis of eight populations showed that the paired Fst values were between 0.011 (SS/WH) and 0.138 (YB/NJ), indicating low to moderate differentiation among different populations and the genetic differentiation between YB and NJ population was the most significant (Table 9). The gene flow (Nm) among eight populations ranged from 1.557 (YB/NJ) to 23.383 (SS/WH), indicating that the gene flow among populations was at a low or medium level, with the largest gene flow between SS population and other populations (1.702~23.383) and the smallest gene flow between YB population and other populations (1.702~2.071).

**Figure 3:**
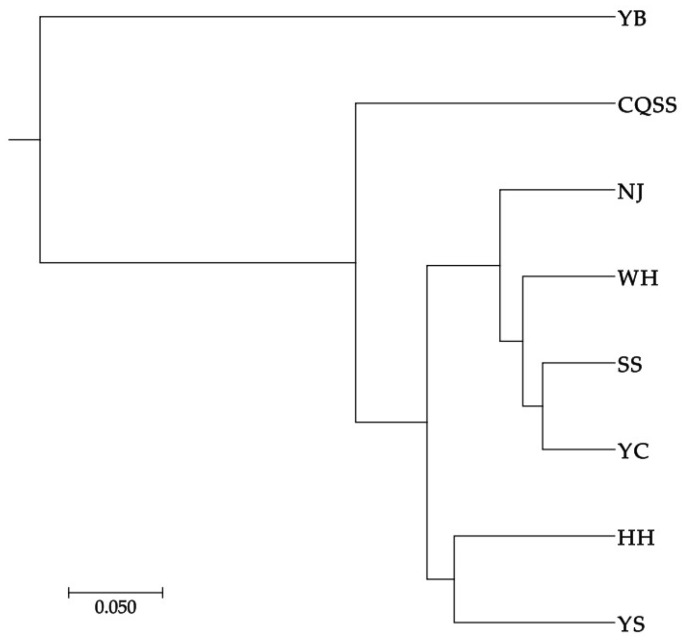
UPGMA phylogram for eight populations of *Hemibagrus macropterus* based on SSRs. YB, Yibin; YC, Yichang; SS, Shishou; WH, Wuhan; NJ, Nanjing; HH, Huaihua; YS, Yangshuo; CQSS, Stock seed.

The maximum genetic distance among eight populations was 0.827 (YB/WH) and the minimum genetic distance was 0.226 (SS/WH) (Table 10). The unweighted paired average method (UPGMA) based on Nei genetic distance was used for cluster analysis. The UPGMA phylogram showed that there were two branches, YB population was located in one independent branch, and the other seven populations were located in another independent branch (Figure 3).

**Figure 4:**
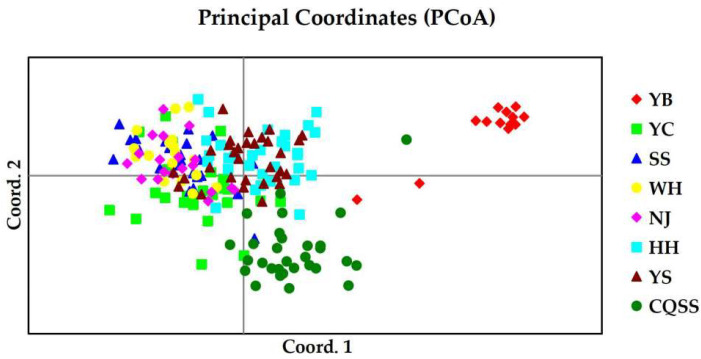
PCoA of eight populations of *Hemibagrus macropterus* based on SSRs. YB, Yibin; YC, Yichang; SS, Shishou; WH, Wuhan; NJ, Nanjing; HH, Huaihua; YS, Yangshuo; CQSS, Stock seed.

**Figure 5:**
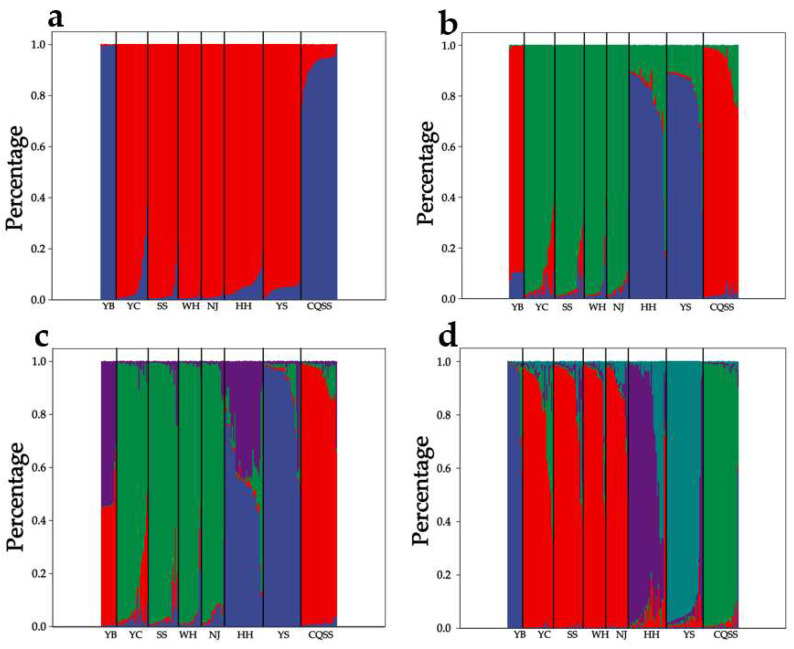
(**a**) Structural diagram of *Hemibagrus macropterus* for K = 2; (**b**) Structural diagram of *Hemibagrus macropterus* for K = 3; (**c**) Structural diagram of *Hemibagrus macropterus* for K = 4; (**d**) Structural diagram of *Hemibagrus macropterus* for K = 5. Different colors represent different clustering subgroups. YB, Yibin; YC, Yichang; SS, Shishou; WH, Wuhan; NJ, Nanjing; HH, Huaihua; YS, Yangshuo; CQSS, Stock seed.

The result of principal coordinate analysis (PCoA) was consistent with the result of UPGMA phylogram. YB population formed a single cluster, while other populations formed the other cluster (Figure 4). The results of molecular variance analysis (AMOVA) showed that 7% of the genetic variation occurred between populations, 3% among individuals, and 90% within individuals, and the genetic variation within individuals was the main source of the total variation (Table 11). To further understand the clustering situation of largefin longbarbel catfish, each individual was analyzed based on likelihood function. The value of ∆K reached the maximum at K = 5, indicating that all individuals were most likely to be classified into five clusters (Figure 5 and Figure S1). Among them, the populations in the middle and lower reaches of the Yangtze River were clustered into a subgroup, and the other four populations were each a subgroup.

### 3.3. Demographic History

**Figure 6:**
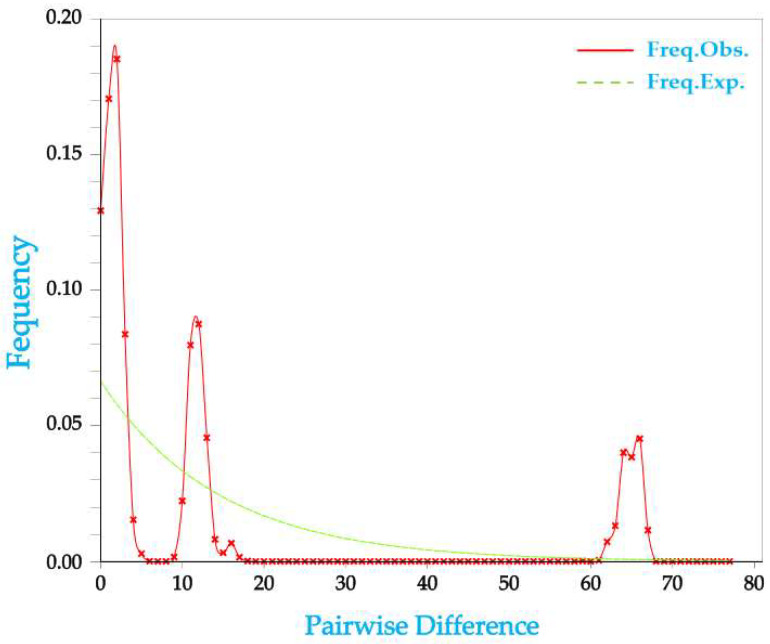
Mismatch distribution of *Cytb* of *Hemibagrus macropterus*.

When the seven wild populations were regarded as a whole for neutral test, Tajima’s D was −0.52814 (*p* > 0.1) and Fu and Li’s D* was 0.56938 (*p* > 0.1), both of which were small and had no statistical significance, indicating that the *Cytb* sequence of largefin longbarbel catfish followed the neutral model in evolution. Meanwhile, mismatch distribution analysis showed multi-peaks, which indicated that the wild largefin longbarbel catfish was in a state of dynamic balance and had not experienced population bottleneck or population expansion recently (Figure 6).

## 4. Discussion

Understanding the level of genetic diversity within a species is crucial for assessing its adaptive potential in response to environmental changes [42]. It also serves as a prerequisite for the sustainable utilization of germplasm resources [27]. Information regarding population structure offers valuable scientific insights for the management and conservation of different populations and contributes to the study of biogeography [28]. In this study, high level of genetic diversity and a low to moderate level of genetic differentiation among populations of the largefin longbarbel catfish were revealed.

### 4.1. Genetic Diversity

Haplotype diversity and nucleotide diversity serve as crucial metrics for assessing the genetic variation in mitochondrial DNA, providing insights into the level of genetic diversity based on their numerical values [42]. A population is deemed to have low genetic diversity when its haplotype diversity is below 0.5 and its nucleotide diversity index is less than 0.005 [43]. In the present study, the haplotype diversity and nucleotide diversity of eight populations of largefin longbarbel catfish were found to be 0.853 and 0.0127, respectively, suggesting a high level of genetic diversity. When compared to the genetic diversity of other aquatic organisms based on the *Cytb* gene, such as *Leiocassis longirostris* (Hd = 0.5417) [44], *Megalobrama amblycephala* (Hd = 0.768) [42], and *Pangasius bocourti* (Hd = 0.742) [45], the genetic diversity indices of the largefin longbarbel catfish are relatively high. Furthermore, the 14 microsatellite loci employed in this study exhibited a high degree of polymorphism (PIC > 0.5), underscoring their substantial utility for genetic diversity analysis [46]. In the context of microsatellite sequence analysis, heterozygosity serves as a crucial parameter for assessing population genetic diversity [43]. Compared to the heterozygosity observed in microsatellites of other freshwater fish species in China, the largefin longbarbel catfish exhibited higher observed alleles (Na = 18.500) and observed heterozygosity (Ho = 0.761) than *Hypophthalmichthys nobilis* (Na = 11.333, Ho = 0.718) [46], *Mylopharyngodon piceus* (Na = 7.6, Ho = 0.744) [47], and *Hypophthalmichthys molitrix* (Na = 11.857, Ho = 0.521) [48]. These findings suggest that the largefin longbarbel catfish currently maintains a high level of genetic diversity, corroborating the results obtained from *Cytb* gene sequence analysis. Despite the significant decline in wild populations of the largefin longbarbel catfish in recent decades, the species retains substantial genetic diversity, indicating its high adaptive potential and relatively low danger of reduced genetic diversity in researched populations, including the stock population (CQSS).

Among the eight populations of *Hemibagrus macropterus*, the YB population exhibited the lowest genetic diversity, potentially attributable to its relatively small sample size of 13 individuals compared to other populations. The genetic diversity indices of a population are often correlated with sample size, with larger samples generally exhibiting higher genetic diversity [49]. Historically, the largefin longbarbel catfish constituted a significant portion of the catch in the upper reaches of the Yangtze River, contributing to a substantial yield [5]. Overfishing may therefore be a contributing factor to the reduced genetic diversity observed in the YB population. Conversely, the HH population demonstrated the highest level of genetic diversity, which may be linked to its proximity to the National Aquatic Germplasm of Largefin longbarbel catfish Conservation Zone in Yuanjiang. Scientific management and protection system in the conservation zone, including protection facilities construction, resource protection, and ecological restoration, may contribute to the high genetic diversity of HH population.

### 4.2. Genetic Structure

The genetic differentiation coefficient (Fst) serves as a crucial index for assessing the extent of genetic divergence among populations [43]. Analyses of paired genetic differentiation coefficients derived from the *Cytb* gene and microsatellite markers indicate significant genetic differentiation between the YB population and the other seven populations. The construction of the Gezhouba and Three Gorges Dams has isolated the upper reaches of the Yangtze River, creating a distinct environment that likely contributes to this genetic divergence. Studies on the genetic structure of other Bagridae fishes, such as *Pelteobagrus fulvidraco* [50], *Hemibagrus guttatus* [51], and *Leiocassis longirostris* [52], further corroborate the substantial impact of these dams. Additionally, historical evidence suggests that the upper and mid-lower reaches of the Yangtze River were once separate rivers, which may have also influenced the genetic structure of the largefin longbarbel catfish [28]. However, the small sample size of YB population may affect the representativeness of haplotype distribution, thus adversely affecting the analysis results of population structure [49,53], so a larger sample size is necessary in future studies.

The haplotype network analysis based on the *Cytb* gene and the structural diagrams derived from microsatellite data both indicated that the genetic relationship between the HH population and the other three populations in the middle reaches of the Yangtze River is relatively distant. This genetic divergence may be attributed to the ecological preferences of the largefin longbarbel catfish. The HH population was sampled from Yuanjiang, a tributary of the Yangtze River that traverses Dongting Lake before joining the main river. Given that the largefin longbarbel catfish typically inhabits rapid-flow environments [28], Dongting Lake likely acts as a barrier to its dispersal [54], potentially increasing the genetic distance between the HH population and other populations in the middle reaches of the Yangtze River.

It is noteworthy that the genetic differentiation between the YS population in the Pearl River basin and populations in the mid-lower reaches of the Yangtze River is minimal. The YS population was sampled from the Lijiang River, a tributary of the Pearl River. Since 214 BC, when the Lingqu Canal was constructed and opened for navigation, the Lijiang River and the Yangtze River basins have been interconnected [55,56], potentially facilitating gene flow among largefin longbarbel catfish across these basins [28]. Furthermore, as a species of commercial significance, modern trade practices could facilitate genetic exchange between the Yangtze River and Pearl River basins. Given the relatively close proximity between HH and YS, the impact of contemporary trade may be more pronounced. Additionally, certain individuals from the CQSS population have been utilized for artificial release in the Yangtze River basin, potentially resulting in introgression from the CQSS population into the YB and YC populations.

## 5. Conclusions

In this study, the genetic background of both wild and stock seed populations was analyzed using mtDNA and SSRs, providing a comprehensive examination of the genetic diversity and structure across different populations. Our findings indicate that the largefin longbarbel catfish exhibits high genetic diversity, with significant genetic differentiation observed between the YB population and other populations. This may suggest that the population in the upper reaches of the Yangtze River should be managed and conserved as a management unit. Additionally, these findings offer scientific guidance for selecting parent stock for artificial propagation. For future research, it is essential to incorporate a greater number of sampling locations and increase the sample size. Additionally, the genomic data of the largefin longbarbel catfish facilitate the application of genomic approaches, including SNPs and structural variation.

## Figures and Tables

**Table 1:** Information on *Hemibagrus macropterus* samples.

Location	Population Name	Sample Size	Geographic Location	Collection Date
Upper reaches of Yangtze River	Yibin (YB)	13	Sichuan (28.7669° N, 104.6279° E)	June 2022
Middle reaches of Yangtze River	Yichang (YC)	26	Sichuan (30.4152° N, 111.8944° E)	June 2023
	Shishou (SS)	25	Hubei (29.7382° N, 112.3955° E)	October 2021
	Wuhan (WH)	19	Hubei (30.6827° N, 114.5270° E)	November 2021
Lower reaches of Yangtze River	Nanjing (NJ)	19	Jiangsu (32.0646° N, 118.8024° E)	June 2023
Xiang Jiang River (Yangtze River tributary)	Huaihua (HH)	32	Hunan (27.3469° N, 109.1798° E)	April 2024
Li Jiang River (Pearl River tributary)	Yangshuo (YS)	31	Guangxi (24.7784° N, 110.4965° E)	May 2024
Chongqing city	Stock seed (CQSS)	30	Chongqing (29.9138° N, 107.2414° E)	March 2023

**Table 2:** Frequency distribution of haplotypes in eight populations of *Hemibagrus macropterus*.

Haplotype	Location							
	YB	YC	SS	WH	NJ	HH	YS	CQSS
Hap_1	12	0	0	0	0	0	0	0
Hap_2	1	9	6	0	1	0	5	16
Hap_3	0	11	12	10	7	0	0	12
Hap_4	0	1	2	0	1	0	0	0
Hap_5	0	2	0	0	0	0	0	2
Hap_6	0	1	2	5	2	2	23	0
Hap_7	0	1	0	0	0	0	0	0
Hap_8	0	1	0	0	0	0	0	0
Hap_9	0	0	2	0	1	0	0	0
Hap_10	0	0	1	0	0	0	0	0
Hap_11	0	0	0	3	2	0	0	0
Hap_12	0	0	0	1	1	0	0	0
Hap_13	0	0	0	0	1	0	0	0
Hap_14	0	0	0	0	2	0	0	0
Hap_15	0	0	0	0	1	0	0	0
Hap_16	0	0	0	0	0	10	0	0
Hap_17	0	0	0	0	0	2	0	0
Hap_18	0	0	0	0	0	1	0	0
Hap_19	0	0	0	0	0	2	0	0
Hap_20	0	0	0	0	0	1	0	0
Hap_21	0	0	0	0	0	4	0	0
Hap_22	0	0	0	0	0	1	0	0
Hap_23	0	0	0	0	0	1	0	0
Hap_24	0	0	0	0	0	1	0	0
Hap_25	0	0	0	0	0	1	0	0
Hap_26	0	0	0	0	0	2	0	0
Hap_27	0	0	0	0	0	1	0	0
Hap_28	0	0	0	0	0	1	0	0
Hap_29	0	0	0	0	0	2	0	0
Hap_30	0	0	0	0	0	0	2	0
Hap_31	0	0	0	0	0	0	1	0

**Table 3:** Genetic diversity in eight populations of *Hemibagrus macropterus* based on *Cytb*.

Population	N	h	Hd	π
YB	13	2	0.154	0.0100
YC	26	7	0.717	0.0108
SS	25	6	0.720	0.0013
WH	19	4	0.661	0.0015
NJ	19	10	0.860	0.0035
HH	32	15	0.887	0.0048
YS	31	4	0.432	0.0005
CQSS	30	3	0.570	0.0085
Total	195	31	0.853	0.0127

N: Number of individuals; h: Number of haplotypes; Hd: Haplotype diversity; π: Nucleotide diversity.

**Table 4:** Information of 14 SSR loci in *Hemibagrus macropterus*.

Locus	Forward Primer	Reverse Primer	Allele Ranges (bp)
Hem002	GAGATCGAGGAGAGCGGGAA	CACCACCTCCGTCATGTTCT	314–334
Hem003	TCGGTGTTTGTTGTCCGAAA	ACGCGGGTAGTAGTAGTGGT	223–248
Hem004	TATGGAGTTGTCCCGCCCTA	CATGCGCAGTAGAGGGAGAT	152–191
Hem005	CAGCTCCGACTCCATGACTG	TAAGCTGCAATGCACCGTTG	297–320
Hem010	GTGCACTGATTCAGCTCCCT	CCAACCCTAGTCCTGCAGTG	259–290
Hem012	GTACTTGCTCTTGACGCTGC	GCGACCTCACGGTTAGAACA	222–273
Hem015	TTCAACAGGTCAGGCTTGCA	GTCCGAACACCAACCGGTAT	251–270
Hem022	CTGTGGTGCCTGAGAGATGG	ACCAACACCATGCTTCACCA	177–202
Hem032	CAAACCCTGGAGTCCTGTCC	ATGTGCTTCACGGAGCTTGT	278–314
Hem036	ACCTGGTTGATGACTGCTGG	TGATGCAGTCTTCGCAGTGT	261–283
Hem041	GAAGCGGACAGTGATGACCA	GCTCAACTTCAGCCTGGTGA	256–291
Hem046	TTGTGCCCTGTGATAGCCTG	CCAGCCAGGGAGCAAACTTA	181–287
Hem047	ACCTCACTGTTCCTGCAGTC	GGGAGAAGTGAGGCAAGAGG	244–267
Hem048	AGGGTGATGTGGAAGGACCT	CGACTACCAGCTACCACGTC	233–281

**Table 5:** Genetic variability at 14 SSR loci in 195 individuals of *Hemibagrus macropterus*.

Locus	Na	Ne	I	Ho	He	F	PIC	Prob	Signif
Hem002	9	2.599	1.256	0.495	0.615	0.196	0.557	0.153	ns
Hem003	18	9.902	2.500	0.874	0.899	0.027	0.891	0.000	***
Hem004	18	11.491	2.579	0.747	0.913	0.181	0.906	0.000	***
Hem005	17	6.850	2.247	0.774	0.854	0.094	0.840	0.716	ns
Hem010	19	4.349	1.889	0.682	0.770	0.114	0.743	0.017	*
Hem012	18	7.980	2.346	0.846	0.875	0.033	0.863	0.999	ns
Hem015	15	4.596	1.868	0.753	0.782	0.038	0.755	0.054	ns
Hem022	16	8.641	2.322	0.794	0.884	0.102	0.873	0.588	ns
Hem032	20	10.234	2.557	0.788	0.902	0.127	0.895	0.244	ns
Hem036	14	4.525	1.844	0.696	0.779	0.107	0.754	0.882	ns
Hem041	44	13.787	3.026	0.907	0.927	0.022	0.923	0.836	ns
Hem046	17	2.737	1.361	0.644	0.635	−0.014	0.574	0.011	*
Hem047	15	6.878	2.157	0.722	0.855	0.156	0.839	0.103	ns
Hem048	19	10.260	2.504	0.928	0.903	−0.028	0.894	0.393	ns
Mean	18.500	7.488	2.175	0.761	0.828	0.083	0.808		
St Dev	7.842	3.426	0.488	0.114	0.100	0.071	0.118		

Na, observed alleles; Ne, effective allele; I, Shannon index; Ho, observed heterozygosity; He, expected heterozygosity; F, fixation index; PIC, polymorphic information index; Prob, probability of genotype frequency randomly deviating from Hardy–Weinberg expectation; Signif, significance (ns represents not significant, the population conforms to HWE; * represents significant difference *p* < 0.05 and *** represents significant difference *p* < 0.001).

**Table 6:** Genetic diversity of 14 SSR loci in eight populations of *Hemibagrus macropterus*.

Population		Na	Ne	I	Ho	He	F
YB	Mean	6.786	4.293	1.539	0.697	0.713	0.013
	SE	0.712	0.463	0.132	0.049	0.046	0.039
YC	Mean	9.357	5.062	1.764	0.734	0.760	0.043
	SE	0.929	0.554	0.126	0.048	0.032	0.040
SS	Mean	8.714	5.145	1.744	0.783	0.765	−0.020
	SE	0.766	0.579	0.120	0.045	0.030	0.042
WH	Mean	7.929	4.904	1.712	0.789	0.765	−0.035
	SE	0.675	0.472	0.110	0.036	0.027	0.039
NJ	Mean	7.714	4.870	1.624	0.711	0.736	0.022
	SE	0.854	0.723	0.139	0.030	0.034	0.036
HH	Mean	11.857	7.457	2.089	0.830	0.831	0.001
	SE	1.346	0.891	0.128	0.029	0.025	0.021
YS	Mean	10.786	5.947	1.904	0.789	0.793	0.006
	SE	0.973	0.720	0.123	0.034	0.028	0.023
CQSS	Mean	7.857	4.455	1.568	0.706	0.706	0.028
	SE	0.824	0.524	0.142	0.063	0.050	0.052

Na, observed alleles; Ne, effective allele; I, Shannon index; Ho, observed heterozygosity; He, expected heterozygosity; F, fixation index.

**Table 7:** Pairwise estimates of genetic distance (above the diagonal) and Fst (below the diagonal) for eight populations of *Hemibagrus macropterus* based on *Cytb*.

Population	YB	YC	SS	WH	NJ	HH	YS	CQSS
YB		0.0569	0.0612	0.0611	0.0614	0.0624	0.0622	0.0573
YC	0.8135		0.0064	0.0065	0.0074	0.0133	0.0066	0.0094
SS	0.9053	0.0369		0.0014	0.0024	0.0096	0.0015	0.0052
WH	0.9031	0.0382	−0.0002		0.0025	0.0098	0.0016	0.0053
NJ	0.8867	0.0168	−0.0012	−0.0148		0.0102	0.0025	0.0062
HH	0.8780	0.4167	0.6831	0.6762	0.5920		0.0094	0.0127
YS	0.9137	0.1398	0.3805	0.3541	0.1908	0.7195		0.0055
CQSS	0.8355	−0.0333	0.0381	0.0398	0.0168	0.4824	0.1732	

**Table 8:** Analysis of molecular variance (AMOVA) test on *Cytb* in eight populations of *Hemibagrus macropterus*.

Source	d.f.	SS	VC	%	F
Among populations	7	821.139	4.75671 Va	65.65	FST = 0.65645
Within populations	187	465.517	2.48940 Vb	34.35	
Total	194	1286.656	7.24611		

Source, source of variation; d.f., degree of freedom; SS, sum of squares; VC, Variance components; %, percentage of variation; F, fixation index.

**Table 9:** The pairwise Fst value (below the diagonal) and number of migrants (Nm, above the diagonal) among eight populations of *Hemibagrus macropterus* based on SSRs.

	YB	YC	SS	WH	NJ	HH	YS	CQSS
YB	-	1.728	1.702	1.656	1.557	2.071	1.887	1.714
YC	0.126	-	18.279	15.648	16.312	8.115	7.332	6.365
SS	0.128	0.013	-	23.383	16.669	8.626	6.530	3.968
WH	0.131	0.016	0.011	-	12.099	8.257	6.847	3.593
NJ	0.138	0.015	0.015	0.020	-	7.770	7.410	3.672
HH	0.108	0.030	0.028	0.029	0.031	-	11.969	4.388
YS	0.117	0.033	0.037	0.035	0.033	0.020	-	3.968
CQSS	0.127	0.038	0.059	0.065	0.064	0.054	0.059	-

**Table 10:** Genetic distance among eight populations of *Hemibagrus macropterus* based on SSRs.

	YB	YC	SS	WH	NJ	HH	YS	CQSS
YB								
YC	0.772							
SS	0.796	0.230						
WH	0.827	0.258	0.226					
NJ	0.802	0.257	0.257	0.302				
HH	0.775	0.373	0.372	0.393	0.400			
YS	0.794	0.406	0.428	0.414	0.389	0.361		
CQSS	0.715	0.398	0.474	0.519	0.482	0.512	0.528	

**Table 11:** AMOVA of eight populations of *Hemibagrus macropterus* based on SSRs.

Source	df	TV	MS	Est. Var.	%
Among Populations	7	179.013	25.573	0.412	7%
Among Individuals	187	1063.128	5.685	0.205	3%
Within Individuals	195	1028.500	5.274	5.274	90%
Total	389	2270.641		5.892	100%

Source, source of variation; df, degree of freedom; TV, total variance; MS, mean square error; Est. Var., estimated difference value; %, percentage of variation.

## Data Availability

The data are contained in the article.

## Supplementary Materials

The following supporting information can be downloaded at: https://www.mdpi.com/article/10.3390/ani15060770/s1, Figure S1: K value change diagram of *Hemibagrus macropterus* based on SSR.
