# Supplementary figures and images for: Genetic Diversity and Population Structure of Largefin Longbarbel Catfish (Hemibagrus macropterus) Inferred by mtDNA and Microsatellite DNA Markers
Source: Animals (Basel). 2025 Mar 8;15(6):770. doi: 10.3390/ani15060770 (PMC11939597; doi:10.3390/ani15060770)

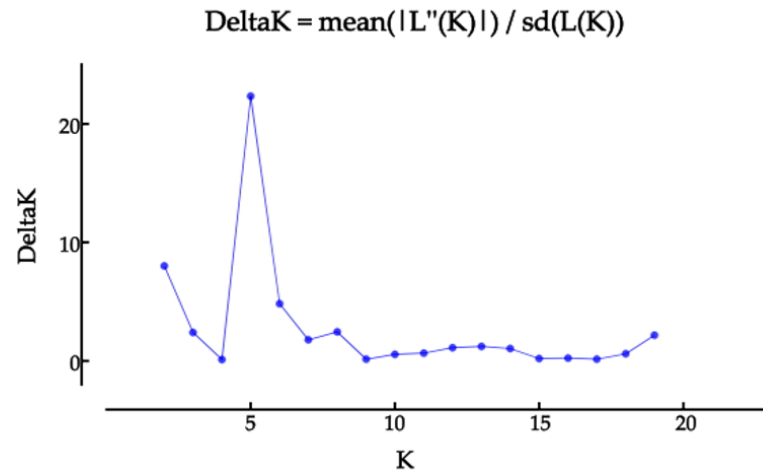

**Figure S1.** K value change diagram of *Hemibagrus macropterus* based on SSR.

Supplement: Supplementary file 1 [file animals-15-00770-s001.zip › animals-3470426-supplementary.pdf]
